# Barriers to appropriate prescribing in older adults with multimorbidity: A modified Delphi study

**DOI:** 10.1002/agm2.12169

**Published:** 2021-07-20

**Authors:** Penny Lun, Jia Ying Tang, Jia Qi Lee, Keng Teng Tan, Wendy Ang, Yew Yoong Ding

**Affiliations:** ^1^ Geriatric Education And Research Institute Limited Singapore Singapore; ^2^ Department of Pharmacy Tan Tock Seng Hospital Singapore Singapore; ^3^ Department of Pharmacy Changi General Hospital Singapore Singapore; ^4^ Geriatric Medicine Tan Tock Seng Hospital Singapore Singapore

**Keywords:** aged, ambulatory care, multimorbidity, polypharmacy, practice patterns (physicians’)

## Abstract

**Objectives:**

We aimed to understand the barriers experienced by physicians when prescribing for older adults with multimorbidity in specialist outpatient clinics in Singapore.

**Methods:**

A modified Delphi study was conducted via email with 20 panel experts in the field of geriatric medicine. Barriers identified from an earlier scoping review were presented as statements to the panel.

**Results:**

Eleven barrier statements reached consensus with high importance according to the Delphi panel. Of these statements, seven (64%) belong to the domain of *Environmental context and resources* in the Theoretical Domains Framework (TDF), while the remaining barriers belong to the domains of *skills, knowledge, intentions, and professional/social role and identity*. The barriers are further linked to intervention functions in the Behaviour Change Wheel (BCW).

**Conclusion:**

Linking the TDF domains to intervention functions revealed strategic directions for the development of an intervention to address the barriers and optimize prescribing.

## Graphical abstract

Eleven barrier statements reached consensus with high importance according to the Delphi panel. Linking the barriers to Theoretical Domains Framework and intervention functions revealed strategic directions for the development of an intervention to address the barriers and optimize prescribing.

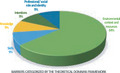

## 1 BACKGROUND

Potentially inappropriate prescribing (PIP) in older adults is a rising concern around the world, as life expectancies are increasing and people are living longer with multimorbidity.^1^ Inappropriate prescribing can be partly attributed to polypharmacy, which is commonly defined as taking five or more medications daily.^2^ Although there is variability in the definition of polypharmacy in the literature, polypharmacy is unavoidable among older adults as they are more likely to have comorbid or multimorbid conditions. Polypharmacy increases the risk of one being prescribed inappropriate medications with risks outweighing their benefits, which could result in adverse drug events, drug interactions, decline in functional status, cognitive impairment, falls, urinary incontinence, and reduced nutritional status.^3^ A systematic review on the prevalence of potentially inappropriate medication use in older inpatients with or without cognitive impairment found the range to be from 0.6% to 88.5% when using clinical tools such as Beers criteria and Screening Tool of Older Persons’ Prescriptions (STOPP).^4^ With trends in polypharmacy and PIP expected to continue rising among older adults,^1^, ^5^ it is crucial to understand factors leading to PIP, which would provide insights into interventions that could reverse those trends.

Many factors contribute to the issue of inappropriate prescribing. Some of the wider, systemic issues include the lack of research in patients with multimorbity,^1^ and exclusion of older adults in general from clinical trials.^5^ In addition, most clinical guidelines are based on single diseases and offer no clear guidance for application in multimorbidity.^1^ As a result, these wider systemic issues manifest in day‐to‐day clinical practice as barriers, with the lack of evidence‐based knowledge to inform practice. A previous scoping review identified barriers to effective prescribing among older adults with multimorbidity at the physician‐related, patient‐related, and health‐system‐related levels.^6^ These barriers were mapped onto the Theoretical Domains Framework (TDF), which is an evidence‐based implementation framework that identifies factors that impact behavior change.^7^, ^8^ It entails 14 validated domains that are based on theories of change and their constructs.^8^ The TDF domains could in turn be linked to intervention functions in the Behaviour Change Wheel (BCW) and their associated behavior change techniques^9^ to facilitate the translation of knowledge into practical implementation. As these findings were mostly based on studies conducted in Europe and in other countries with differing health systems and cultures, we needed to explore if those barriers exist in Singapore's context.

Hence, the primary aim of this Delphi study is to identify key barriers to appropriate prescribing for older adults in the outpatient care setting in Singapore. The secondary aim is to link the identified barriers with their already mapped TDF domains to the intervention functions in the BCW. This information will provide the evidence base to guide clinical practice and policy improvements through the development of an intervention prototype that aims to optimize prescribing for older adults with multimorbidity.

## 2 METHODS

### 2.1 Study design

The Delphi technique is commonly used to solicit the opinions of experts and achieve group consensus on a subject matter through a series of structured iterative questionnaires.^10^ Unlike traditional Delphi studies, the modified Delphi technique adopted for this study utilizes preexisting literature to develop the initial questionnaire, rather than starting the first round with open‐ended questions.^10^

### 2.2 Panel participants

Practicing geriatricians with at least three years of post‐professional qualifications in three of Singapore's public hospitals were invited to participate in the study. As there are no set standards on the number of panelists in a Delphi Study and having 10–15 experts with homogenous background is considered appropriate,^10^ we planned to enroll 20 participants to account for chances of attrition. Email invitations were sent to potential participants with an explanation on the purpose of the study, brief results from our scoping review on barriers to effective prescribing, the Delphi process, and the survey period. The invitation continued until we enrolled 20 participants and obtained their informed consent. Ethical approval of the study was granted by the National Health Group Domain Specific Review Board, Singapore (NHG DSRB Ref:2019/00521).

### 2.3 Modified Delphi rounds and the process

This study consisted of two rounds of questionnaires. Statements presented were formulated based on findings from a previous scoping review.^6^ The barriers identified in that study were mapped onto 10 of the 14 domains in the Theoretical Domains Framework: *knowledge; skills; social/professional roles and identity; beliefs about capability; beliefs about consequences; intentions; memory, attention and decision process; environmental contexts and resources; social influences; emotions*.^6^ The barriers were further divided based on stakeholders influencing the prescribing process, either directly or indirectly: physician, patient, or healthcare system at large.^6^ Most barriers were categorized under the physician perspective. In the current study, we reviewed and consolidated similar barriers, when appropriate, resulting in a total of 98 barrier statements for the round 1 questionnaire. Participants were asked to rate the importance of the barriers as factors impacting their prescribing process. In addition to the rating, a comment box was added below each statement for participants who wished to comment or explain their decisions further.

Round 2 of the Delphi questionnaires contained statements that have low group agreement, with some minor refinement based on participants' corresponding comments. Prior to the start of round 2, participants also received formal feedback from round 1 results, comprising the group's median rating of each statement, as well as their respective ratings. The purpose of providing feedback is to create an opportunity for the participants to review and reconsider their stance on the statements,^10^ which in our study, were those that did not reach consensus in round 1.

### 2.4 Defining consensus

A 7‐point Likert scale was used by participants to rate their level of agreement with each statement: 1‐ Not at all, 2‐Low, 3‐Slightly, 4‐Neutral, 5‐ Moderately, 6‐Very, and 7‐ Extremely. A priori criteria adapted from previous studies^11^, ^12^ were used to define consensus, with median and interquartile range used as measures of central tendency and dispersion, respectively.^13^ Only those statements that had low group agreement in round 1 were rerated in round 2. The following criteria were as follows:

- Median ≥6 and IQR ≤1 = High group agreement on being very and extremely important →Item is included,
- Median <6 and IQR ≤1 = High group agreement on being of moderate or low importance →Item is removed,
- IQR >1 = Low group agreement on level of importance (nonconsensus) →Item is refined and continued to round 2 for rating.

Due to the large number of barriers derived from the scoping review results,^6^ we made an a priori decision to only consider barrier statements that fulfill the criteria for high importance in the subsequent intervention design. Hence, the statements with consensus on moderate to low importance and statements that did not reach consensus were not analyzed.

### 2.5 Mapping barriers onto intervention functions

Barriers that were regarded as highly important by the panel were mapped to intervention functions to characterize the types of intervention elements that would best address them.

## 3 RESULTS

### 3.1 Delphi rounds

**FIGURE 1:**
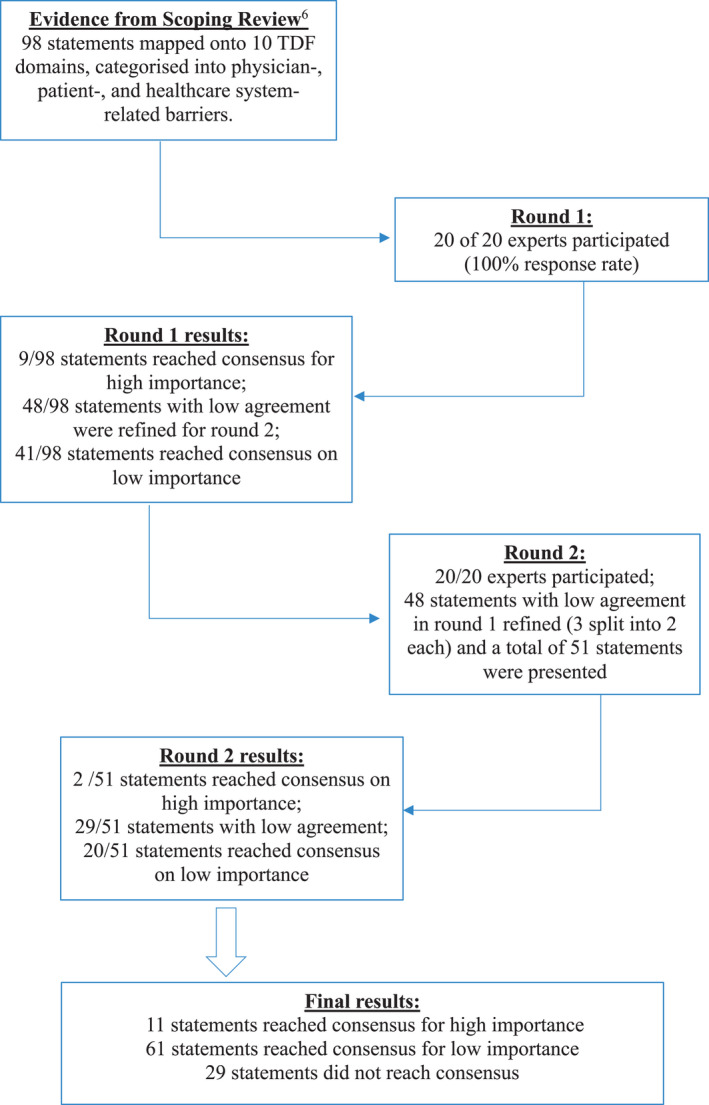
Flowchart of the modified Delphi process.

The Delphi study took place from September 2019 to December 2019. All 20 participants responded to both rounds of the survey. Among the participants from the Geriatric Medicine clinics of three public hospitals, 10 were males (50%) and 10 were females (50%). Among them, 17 (85%) were qualified geriatricians while the remaining 3 were senior resident physicians with extensive experience in geriatric medicine. Of the 98 statements that were presented in round 1 of the survey, 9 reached consensus, while 41 were deemed to be of lower importance and were not pursued further. The remaining 48 statements that did not reach consensus (IQR > 1) were refined and included in the second round of the survey for repeat rating. Among these 48 statements, three that belonged to the domain of *skills (physician‐related)* were split into two statements each to further clarify the concepts. Out of the 51 presented statements in round 2, two reached consensus with high importance, whereas 20 reached consensus for moderate to low importance. Consensus was not reached for the remaining 29 statements. Figure 1 shows the flowchart of the Delphi process and results.

### 3.2 Consensus barrier statements with high importance

Based on our a priori criteria, statements with an interquartile range (IQR) of 1 or less and median of 6 or above were considered to have reached consensus for high importance. Accordingly, 11 statements were identified as highly important barriers to appropriate prescribing as shown in Table 1. Of these statements, seven (64%) were previously mapped onto the domain *environmental context and resources* in TDF,^6^ which represented the broader healthcare system perspective. This cluster of barriers shines the light on challenges encountered in specialist outpatient clinics, where older adults with multimorbidity receives medications from multiple prescribers. The current outpatient clinical environment is fragmented with having no single coordinating physician and the lack of communication between various providers. Moreover, there is lack of ownership to assume responsibility for optimizing the patient's individual care plans. The paucity of evidence‐based recommendations for older adults with multiple chronic illnesses due to the exclusion of such patients in clinical trials exacerbates the challenge of appropriate prescribing. Some of these points are evident from comments provided by the panel:

Seen by many doctors therefore one individual does not take the overall management of the patient (Participant 02, Site 1).

As one physician lacks the responsibility or the oversight of the patient’s care, medications may be started or stopped inappropriately, especially if the patient is not involved in the decision‐making process (Participant 17, Site 2).

Not easy to see why certain medications started/stopped especially if done so in another institution/GP/private hospital (Participant 09, Site 3).

**TABLE 1:** Consensus barrier statements with high importance

TDF	Statements	Minimum	Median	Maxi‐mum	IQR
Physician‐related barriers					
Skills	Lack of research on older adults with multimorbidity.^26^	Slightly (5%)	Very (60%)	Extremely (15%)	1
Environmental context and resources	Hesitancy in changing medications that have been prescribed in their current dosage for a long period, or when not the original prescriber.^20^	Low (5%)	Very (35%)	Extremely (20%)	1
Intentions	Easier to maintain the status quo rather than interfere with drug regimens in a stable patient.^27^	Neutral (5%)	Very (45%)	Extremely (15%)	1
Professional/social role and identity	Reluctance to interfere with medications that have been prescribed by a colleague or specialist (ie, hesitation in discontinuing medications prescribed by another physician).^21^	Slightly (5%)	Very (45%)	Extremely (15%)	1
Patient‐related Barriers					
Knowledge	Patients do not understand what medications they are taking.^17^	Slightly (5%)	Very (50%)	Extremely (10%)	1
Healthcare System–related Barriers					
Environmental context and resources	Patients follow up with multiple hospitals and receive medications from multiple providers.^22^	Moderately (10%)	Very (55%)	Extremely (35%)	1
	Increased specialization in healthcare (ie, focus on subspecialty‐based care instead of overall management).^28^	Slightly (5%)	Very (50%)	Extremely (20%)	1
	Fragmentation of care, lack of a specific or unified physician to follow up with^23^	Moderately (20%)	Very (55%)	Extremely (25%)	1
	Lack of coordination or communication between transitions and various levels of care across healthcare settings.^21^, ^29^	Slightly (5%)	Very (40%)	Extremely (20%)	1
	Exclusion of multimorbid older adults in clinical trials.^21^	Neutral (20%)	Very (55%)	Very (55%)	1
	Lack of ownership to assume responsibility for optimizing a specific patient's care plans.^24^	Neutral (5%)	Very (65%)	Extremely (25%)	1

One of the barriers identified under the domain *skills (physician‐related)* was a lack of research in older adults with multimorbidity:

Research studies often do not include the elderly patients; hence it is difficult to apply study results/knowledge into this group of patients. This reduces the confidence in appropriately prescribing for the elderly (Participant 06, site 2).

I feel more importance (sic) given to disease management, rather than age‐specific issues and taking into consideration interaction of medication in different age group (Participant 02, site 1).

Although the remaining barriers from the physicians' perspective cluster were mapped onto different domains, namely in *environmental context and resources, intentions,* and *professional/social role and identity*,^6^ they shared similar sentiments in reflecting uncertainty and aloneness in the prescribing decision‐making process. This leads to reluctance in making changes to medications that have been prescribed for long periods or by other physicians. In other words, maintaining the status quo in prescribing is easier. One of the participant's comments sums up the observation:

As patient’s medication list become (sic) more complex, there are many specialty drugs that not all physicians will be familiar with. Hence, there will be hesitancy in changing medications prescribed by another prescriber. Also, sometimes, the patient has a long term rapport with the other prescriber, or has (sic) the impression that they are doing well on the current regimen, thus, patient will be reluctant to have the medication/dosage change (Participant 17, site 2).

Only one barrier identified is related to patients' perspective and that is their lack of knowledge of the medications that they are taking. However, there is indication that the lack of knowledge is due to inadequate communication with the patients:

In the local context, patients are not very well informed of their own medications. They are also not aware of the indications and side effects. Often, medications are started both in inpatient and outpatient settings without their knowledge, and they just take the medications. There is thus lack of meaningful discussion between the healthcare provider and the patient (Participant 17, site 2).

### 3.3 Remaining barrier statements

At the end of the round 2 survey, 61 barriers were deemed to be of lower importance based on our criteria and 29 barrier statements did not reach consensus. Although the results were not further analyzed, the TDF domains found among these statements were: *beliefs about capability; memory, attention, and decision processes; emotion; social influences*; *beliefs about consequences*. The barrier statements with consensus on low importance and nonconsensus can be found in Tables A1 and A2 respectively in the Appendix.

### 3.4 Linking barriers to intervention functions

For our secondary aim, the 11 barrier statements identified as highly important were linked to the intervention functions in the BCW,^9^ via their mapped TDF domains.^6^ Table 2 shows results of the linkage. The Behaviour Change Wheel has its core in capability, opportunity, motivation, and behavior (COM‐B), interacting with each other.^9^ All 9 intervention functions in the BCW were mapped onto the 11 barrier statements. Some TDF domains are linked to more than one intervention function, which means that for those respective domains, there are various ways to address them. For example, the barrier on hesitancy in changing medication, which belongs to the *Environmental context and resources* TDF domain, could be addressed with intervention functions such as training, restriction, environmental restructuring, and enablement. The definitions of intervention functions were adopted from Michie et al. (2014).^9^

**TABLE 2:** Linking the barriers identified to their respective COM‐B, TDF, and intervention functions (adapted from Michie at al., 2014)

Barrier Statements	COM‐B	TDF	Intervention functions
Lack of research on older adults with multimorbidity.^26^	Physical capability	Physical skills	Training
Patients do not understand what medications they are taking.^17^	Psychological capability	Knowledge	Education
Reluctance to interfere with medications that have been prescribed by a colleague or specialist (ie, hesitation in discontinuing medications prescribed by another physician).^21^	Reflective motivation	Professional/social role and identity	Education, Persuasion, Modelling
Easier to maintain the status quo rather than interfere with drug regimens in a stable patient.^27^		Intentions	Education, Persuasion, Incentivization, Coercion, Modelling
Hesitancy in changing medications that have been prescribed in their current dosage for a long period, or when not the original prescriber.^20^	Physical opportunity	Environmental context and resources	Training, Restriction, Environmental Restructuring, Enablement
Patients follow up with multiple hospitals and receive medications from multiple providers.^22^			
Increased specialization in healthcare (ie, focus on subspecialty‐based care instead of overall management).^28^			
Fragmentation of care, lack of a specific or unified physician to follow up with.^23^			
Lack of coordination or communication between transitions and various levels of care across healthcare settings.^21^, ^29^			
Exclusion of multimorbid older adults in clinical trials.^21^			
Lack of ownership to assume responsibility for optimizing a specific patient's care plans.^24^			

## 4 DISCUSSION

Developing an intervention in a dynamic healthcare setting is a complex process that requires a systematic approach. Using the Medical Research Council's framework on developing and evaluating complex interventions, the development phase consists of identifying evidence base and theory for an intervention.^14^ As such, this study forms part of the evidence base for a wider project to develop an intervention to optimize prescribing for older adults with multimorbidity. To our knowledge, this was the first Delphi study conducted to understand barriers to appropriate prescribing experienced by geriatricians in the outpatient clinics of Singapore's public hospitals.

The Delphi panel reviewed a list of barriers that have been identified from a scoping review^6^ of studies conducted in other countries and categorized into physician‐, patient‐, and healthcare system–related perspectives. The 11 top barriers experienced in our local context are consistent with the themes found in the literature. Besides physician‐related factors, patient‐related factors such as their resistance and ambivalence towards changes,^15^ nonadherence with medications and visits,^16^ and lack of understanding of the medications^17^ were found to affect the prescribing process. With respect to wider healthcare system–related factors, work practice and medical culture^15^, ^16^ and difficulty navigating current evidence‐based guidelines specific to single diseases^16^, ^17^, ^18^ were also found to hinder the prescribing process. In addition, prescribing for an older adult with multimorbidity is a complex process, due to a clear knowledge and skills gap,^15^, ^17^, ^19^ as well as the complex interprofessional relationship from having more than one physician involved.^18^ The latter leads to a barrier identified by our Delphi panel on hesitancy and reluctance to change prescriptions by others,^18^, ^20^ which might set a precedence for devolving of responsibility.^15^

Due to the large number of barriers identified from the scoping review,^6^ an arbitrary cutoff point was determined in the current study, as it would be impossible to address all identified barriers with one intervention. It is also unlikely that an intervention could directly address some of the pervasive healthcare system–related problems that require broader system or policy changes. Hence, the advantage of having separate stakeholder perspectives is that they can help point to the barriers that are most feasible to address. It would also disentangle the individual‐level barriers that were tied to system issues, which help direct a way to circumvent those issues.

In terms of translational implications on health practice and policy, one of the barriers identified under the *environmental context and resources* domain is the exclusion of older adults with multimorbidity in clinical trials.^21^ To address at the systems level, the clarion call for inclusion of older adults with multimorbidity in carefully conducted clinical trials is still relevant. That said, a possible training‐related solution at the organizational level is creation of a knowledge base such as encouraging geriatricians to share case studies on complex older adult patients on a regular basis and adding that information to a departmental repository. This would enable both junior and experienced physicians to gain knowledge and skills to optimize prescribing for this complex population, despite the paucity of evidence‐based guidelines.

To address the related barriers of undue focus on specialty‐based care, receipt of care from multiple prescribers,^22^ fragmentation of this care with lack of coordination,^23^ and ultimately, lack of ownership to assume responsibility for individualized care plans,^24^ restructuring the environment by appointing primary coordinating physicians for each patient could be a possible solution to improve prescribing. Likewise, the tendency to maintain the status quo in prescribing due to one's hesitancy in changing medications^20^ and the reluctance to interfere with medications prescribed by other physicians^21^ may also be addressed by the intervention functions of *environmental restructuring* and *enablement,* through medication reviews by on‐site clinical pharmacists. By providing recommendations based on their reviews, pharmacists provide support to the prescribing process, which makes prescribing for this complex group of patients less lonely and uncertain.

Overall, we demonstrated that it is possible to link barriers experienced by physicians to intervention functions via the TDF and BCW framework. The BCW in turn sheds light on behavior change techniques that could be implemented and tested in a feasibility study for a physician‐pharmacist care collaborative multimodal intervention. Following this, the objective is to scale up and adapt an effective intervention to multiple sites for broader implementation across hospital ambulatory care and primary care in Singapore.

### 4.1 Strength and limitations

A major strength of our study is the utilization of the modified Delphi technique to calibrate the previous scoping review findings^6^ to the local context. As opposed to other group consensus methods like focus group discussions or conferences that require face‐to‐face meetings, questionnaires in a Delphi study can be disseminated and completed by the participants independently via an online platform.

On the other hand, our study has limitations that should be acknowledged. We made the a priori decision to stop at two survey rounds, resulting in 29 barrier statements not reaching consensus. Along with consensus statements that were found to be of lower importance, they were excluded from further analysis. This may have led to overlooking barrier statements that are important.^13^ A way to mitigate this risk of overlooking important barriers is to include post‐hoc considerations with justification,^25^ perhaps by considering some of the excluded statements that were close to our set criteria.

## 5 CONCLUSIONS

Barriers to prescribing for older adults with multimorbidity span across physician, patient, and healthcare system levels. While the literature points to similar barriers globally, no previous study has explored this aspect in Singapore, which has a unique health care system. Our modified Delphi study brought consensus to 11 of the barriers identified in the literature, most of which were mapped under the *environmental context and resources* domain in the Theoretical Domains Framework. This framework is linked to the Behaviour Change Wheel, which provides a systematic method to identify evidence‐based intervention strategies. These can then be incorporated into care interventions to optimize prescribing for older adults with multimorbidity.

## CONFLICT OF INTEREST

We have no conflict of interest to declare.

## AUTHOR CONTRIBUTIONS

Penny Lun contributed to the methodology and investigation and drafted the manuscript as the main writer of the study. Tang Jia Ying participated in the investigation, analysis, data curation, and preparation for the draft manuscript. Lee Jia Qi participated in the methodology planning. Tan Keng Teng Tan and Wendy Ang participated in the conceptualization of the study. Ding Yew Yoong is the architect of the concept, the person in charge of the project, and guided the writing of the paper. All authors have reviewed, read, and approved the content of the manuscript.

## Data Availability

The data that supports the findings of this study are available in the supplementary material of this article.

## APPENDIX 1

**TABLE A1:** Consensus Statements with low importance

TDF	Barrier statements	Minimum	Median	Maximum	IQR
Physician‐related					
Knowledge	Lack of awareness of potentially inappropriate medications	Low (5%)	Moderately (35%)	Extremely (10%)	1
	Poor insight into the term and the process of deprescribing	Slightly (10%)	Moderately & Very (30%), (30%)	Extremely (20%)	1
	Lack of up‐to‐date knowledge	Slightly (15%)	Moderately (40%)	Very (15%)	1
	Multimorbidity, potential interactions between diseases and medications	Slightly (10%)	Moderately & Very (30%), (35%)	Extremely (15%)	1
	Polypharmacy, which increases difficulty in rationalizing and deprescribing medications	Slightly (15%)	Moderately & Very (35%), (40%)	Extremely (10%)	1
Skills	Lack of confidence and clinical experience in managing elderly patients	Low (5%)	Moderately (55%)	Very (30%)	1
	Physicians are reluctant to talk to patients about their life expectancy because of difficulty in estimating life expectancy and cultural taboo	Neutral (15%)	Moderately (45%)	Very (40%)	1
	Physicians are reluctant to talk to patients about their life expectancy due to lack of skills to approach the topic or lack of time	Slightly (5%)	Moderately (40%)	Extremely (5%)	1
	Problems with incorporating patients’ prognoses into decisions about therapy appropriateness	Low (5%)	Moderately & Very (35%), (50%)	Very (50%)	1
	Difficulty in communicating risk(s) and benefit(s) to patient/family	Slightly (10%)	Moderately (55%)	Very (25%)	0.5
	*Difficulty in engaging patient/family in a shared decision‐making process	Slightly (11%)	Moderately (63%)	Very (21%)	0
Social/professional role and identity	Risk/fear of conflict or damaging the relationship between various healthcare providers	Low (15%)	Moderately (70%)	Moderately (70%)	1
	Respect for hierarchy	Slightly (5%)	Moderately (45%)	Very (40%)	1
Beliefs about capability	Influence from prescriber's own beliefs, clinical experience and prescribing habits	Neutral (15%)	Moderately (45%)	Extremely (10%)	1
	Respect prescriber's right to autonomy	Low (10%)	Moderately (50%)	Very (5%)	1
	Fear of causing potential harm by deprescribing (eg, fear of withdrawal effects)	Low (10%)	Moderately (70%)	Very (15%)	0
	Fear of damage to reputation, accountability for adverse outcomes, malpractice, or litigation	Low (10%)	Moderately (50%)	Extremely (5%)	1
Intentions	Easier to pile on the recommendations of one guideline onto another instead of prioritizing	Low (10%)	Moderately (45%)	Extremely (5%)	1
	Deferring treatment decisions or changes to the next visit	Slightly (15%)	Neutral & Moderately (35%), (45%)	Very (5%)	1
Memory, attention, and decision processes	Inability to gauge the efficacy/effectiveness of a drug for individual patients	Slightly (10%)	Moderately (45%)	Extremely (10%)	1
	Managing complex drug regimens and side effects	Slightly (5%)	Moderately & Very (45%), (45%)	Extremely (5%)	1
	Ethical concerns around denying treatments	Not at all (5%)	Moderately (50%)	Very (10%)	1
	Limited availability of alternatives to medication	Not at all (5%)	Neutral (50%)	Extremely (5%)	1
Environmental context and resources	Failure to meet the challenge of complex decision making	Low (5%)	Moderately (50%)	Extremely (5%)	1
	Overall clinical uncertainty in elderly patients	Slightly (15%)	Moderately (60%)	Very (15%)	0.5
	Lack of communication between prescribers before adding on new drugs	Slightly (10%)	Moderately & Very (30%), (30%)	Extremely (20%)	1
	Lack of support from secondary/tertiary care for general practitioners managing complex patients	Slightly (10%)	Moderately (45%)	Extremely (10%)	1
	Feeling pressured by guidelines to prescribe medications (including preventive drugs)	Low (5%)	Moderately (55%)	Extremely (5%)	0.5
	Less comfortable in deprescribing guideline‐recommended therapeutic medications (as compared to deprescribing preventive medications) in patients with poor life expectancy	Low (15%)	Moderately (55%)	Extremely (5%)	0.5
	Pressure to adhere to disease‐specific guidelines	Low (5%)	Moderately (45%)	Very (20%)	1
	Lack of time to perform medication reviews during the clinic consultation visit	Slightly (10%)	Moderately (50%)	Extremely (10%)	1
	Competing demands of practice (ie, prioritizing other aspects of care rather than deprescribing)	Neutral (5%)	Moderately (50%)	Extremely (5%)	1
	Limited prescribing support (formularies and computer decision support have limited adaptability and flexibility with multiple conditions)	Low (15%)	Moderately (50%)	Extremely (5%)	1
	Lack of resources to assist family caregivers with challenging symptoms (eg, incontinence)	Low (10%)	Moderately (65%)	Extremely (10%)	0
	Lack of evidence for the use of or discontinuation of specific drugs for older patients (mainly due to exclusion of multimorbid older patients in clinical trials)	Slightly (5%)	Moderately (45%)	Extremely (10%)	1
Emotion	Feeling a sense of fear towards older patients in general owing to their frailty and comorbidities	Not at all (5%)	Moderately (55%)	Very (10%)	1
Patient‐related					
Environmental context and resources	Patients do not inform GPs about their medication intake or side effects	Slightly (5%)	Moderately (55%)	Extremely (10%)	1
	Lack of adherence to medications, or self‐titration of medications	Slightly (20%)	Moderately (45%)	Extremely (10%)	1
	Usage of over‐the‐counter and traditional medications (often without informing the primary coordinating physician)	Slightly (5%)	Moderately (55%)	Very (25%)	0.5
	Nonadherence to visits	Low (5%)	Moderately (60%)	Very (20%)	0
	Choosing to “doctor or pharmacy hop”	Slightly (10%)	Moderately (70%)	Extremely (10%)	0
	Patients are reluctant or disinclined to stop medications that they have used for a long time, resistant to change, poor acceptance of alternatives	Low (5%)	Moderately (40%)	Extremely (10%)	1
	Unrealistic expectations/demands of patients and families	Low (5%)	Moderately (55%)	Extremely (5%)	0.5
	Personal beliefs, demands, and expectations of patient and family about their care and medications	Slightly (10%)	Moderately (45%)	Very (35%)	1
	Discrepancies between the patients’ preferences and best practice recommendations	Slightly (20%)	Moderately (50%)	Very (15%)	1
	Demanding specific medications and when refused, obtaining them from different physicians	Neutral (15%)	Moderately (65%)	Very (20%)	0
Social influences	Patients’ social context and access to healthcare and resources	Low (5%)	Moderately (65%)	Extremely (5%)	0
	Patients who change living or care arrangements may be accompanied by different caregivers to visits, which may result in inconsistent reports from the family and/or lack of continuity of care	Slightly (5%)	Moderately & Very (35%), (40%)	Extremely (10%)	1
	Patients’ socioeconomic status	Low (15%)	Moderately (65%)	Very (5%)	1
Healthcare system–related					
Environmental context and resources	Specialists’ lack of a holistic or geriatric view on elderly patients	Slightly (5%)	Moderately & Very (40%), (45%)	Extremely (5%)	1
	Inadequate documentation	Neutral (10%)	Moderately (45%)	Extremely (15%)	1
	Poor acquisition and documentation of patients’ medication lists	Slightly (10%)	Moderately (50%)	Extremely (15%)	1
	Difficulty in obtaining colleagues’ reasons for prescription	Slightly (5%)	Moderately (55%)	Extremely (5%)	1
	Difficulty in achieving clear overview of the patient's medical treatment	Low (5%)	Moderately (55%)	Extremely (10%)	0.5
	Quality measure‐driven care	Not at all (5%)	Neutral (50%)	Moderately (20%)	1
	Most guidelines suggest adding medications instead of removing them (ie, EBM guidelines contribute to polypharmacy	Low (10%)	Moderately (65%)	Very (15%)	0
	Challenges in implementing guidelines to elderly patients with multimorbidity	Slightly (5%)	Moderately & Very (40%), (40%)	Extremely (10%)	1
	Widespread marketing of medications in mainstream media	Not at all (5%)	Neutral (40%)	Very (5%)	1
	Lack of access to patients’ clinical data (eg, current medication) from other healthcare settings	Slightly (15%)	Moderately (45%)	Extremely (15%)	1
	Lack of access to expert advice and user‐friendly decision support (eg, computer prompts or alerts to notify prescribers of PIMs)	Low (15%)	Moderately (55%)	Moderately (55%)	1

* Only 19 responses for this statement due to missing data.

**TABLE A2:** Nonconsensus barrier statements

TDF	Barrier statements	Minimum	Median	Maximum	IQR
Physician‐related					
Knowledge	Lack of awareness of medication cost	Low (15%)	Moderately (45%)	Very (15%)	2
	Lack of formal education on prescribing for and treatment of the elderly	Low (5%)	Moderately (40%)	Very (35%)	1.5
Skills	Limited applicability of research findings in day‐to‐day clinical work	Slightly (10%)	Moderately (30%)	Extremely (10%)	1.5
Social/professional role and identity	Physicians imposing their own beliefs onto the patient without consideration for the latter (no shared decision making)	Low (5%)	Moderately (50%)	Extremely (5%)	1.5
	Dilemma between economic responsibility for both patients and society	Low (30%)	Neutral (45%)	Very (10%)	2
	Unwillingness to change recommendations from secondary/tertiary care	Not at all (5%)	Moderately (35%)	Extremely (5%)	2
	Varying acceptance of pharmacists’ recommendation	Not at all (5%)	Neutral (40%)	Very (10%)	1.5
Beliefs about consequences	*Fear of “giving up on the patient”	Low (11%)	Moderately (47%)	Extremely (5%)	2
	Viewing the deprescribing process as a risk to be avoided	Low (15%)	Neutral (25%)	Very (10%)	2
Memory, attention, and decision‐making processes	Feeling forced to prescribe	Low (20%)	Slightly & Neutral (30%), (25%)	Moderately (25%)	1.5
Environmental context and resources	Increased risk of ADRs and drug–drug interactions	Low (5%)	Moderately (25%)	Very (30%)	3
	Difficulty in distinguishing between new complaints and medication side effects	Low (10%)	Moderately (45%)	Very (30%)	1.5
	Pressure from guidelines vs individual patient circumstances	Low (20%)	Moderately (50%)	Very (15%)	2
	Uncertainty about patients who may be eligible for a medication review	Low (15%)	Neutral (25%)	Very (15%)	2
	Physicians themselves may be influenced by pharmaceutical drug representatives	Not at all (5%)	Neutral (35%)	Moderately (25%)	2.5
	Lack of access to a pharmacist (to assist with medication review)	Not at all (10%)	Neutral (25%)	Very (5%)	3
	Lack of available tools/strategies to help quantify benefits and harms	Slightly (10%)	Moderately (40%)	Extremely (15%)	2
Social influences	Culture to prescribe more	Not at all (5%)	Neutral (25%)	Extremely (5%)	2
	Lack of peer support (ie, medication review)	Low (25%)	Neutral (30%)	Moderately (35%)	2.5
	Peer influence	Not at all (5%)	Neutral (45%)	Very (10%)	1.5
Emotion	Choosing to maintain the patient–doctor relationship rather than enforce changes or recommendations and threatening that relationship	Not at all (5%)	Neutral & Moderately (20%), (50%)	Moderately (50%)	2.5
Patient‐related					
Environmental context and resources	Unintentional withholding of ADRs because they attribute these to aging rather than side effects of medications	Not at all (10%)	Neutral (20%)	Moderately (35%)	2
	Patients are more likely to report symptoms to hospital specialists rather than GPs	Not at all (5%)	Neutral (40%)	Very (10%)	1.5
	Some patients “love taking medications”	Low (10%)	Moderately (50%)	Very (15%)	1.5
Healthcare system–related					
Environmental context and resources	Data lost in the transition from written notes to electronic prescriptions	Low (5%)	Moderately (50%)	Very (10%)	1.5
	Influences of prescribing policy (perception of managerial meddling and cost cutting)	Not at all (5%)	Neutral (45%)	Very (5%)	2
	Limited options on healthcare organization formularies	Not at all (10%)	Low (45%)	Moderately (5%)	2
	Lack of data for outcomes most important to patients (eg, improvement in pain control	Low (10%)	Moderately (55%)	Extremely (5%)	1.5
	Difficulty in managing direct‐to‐consumer commercials about drugs and their impact on patients	Low (25%)	Neutral (65%)	Moderately (5%)	1.5

* Only 19 responses for this statement due to missing data.
